# Smoker Identity and Its Potential Role in Young Adults’ Smoking Behavior: A Meta-Ethnography

**DOI:** 10.1037/hea0000191

**Published:** 2015-01-26

**Authors:** Ildiko Tombor, Lion Shahab, Aleksandra Herbec, Joanne Neale, Susan Michie, Robert West

**Affiliations:** 1 Cancer Research UK Health Behaviour Research Centre, University College London; 2 Institute of Psychiatry, Psychology and Neuroscience, King’s College London; 3 Department of Clinical, Educational and Health Psychology, University College London; 4 Cancer Research UK Health Behaviour Research Centre, University College London

**Keywords:** smoker identity, smoking cessation, young adults, systematic review, meta-ethnography

## Abstract

***Objective:*** Identity is an important influence on behavior. To identify potential targets for smoking cessation interventions in young adults, we synthesized findings from qualitative studies on smoker identity and potential influences on smoking and smoking cessation. ***Methods:*** A systematic search of 4 electronic databases up to September 19, 2013, was conducted to identify qualitative studies on smoker identity in smokers and ex-smokers aged 16–34. Key concepts were extracted from individual studies and synthesized into higher-order interpretations by following the principles of meta-ethnography. ***Results:*** Seventeen relevant papers were identified. At the highest level of interpretation, we identified 4 types of findings: (a) contributory factors to identity, (b) identity in relation to smoking, (c) contextual and temporal patterning, and (d) behavior in relation to smoking. Contributory factors included the desire to establish aspirational individual and social identities, enact a smoker identity appropriate to the momentary social context, and alter personal nonsmoking rules when consuming alcohol. Smoker identity was multifaceted and incorporated individuals’ defensive rationalizations, and both positive and negative feelings attached to it. Smoker identities took time to develop, were subject to change, and were context dependent. Identity was found to play a role in quit attempts. ***Conclusions:*** Qualitative research into the identity of young adult smokers has established it as a multifaceted phenomenon serving important functions but also involving conflict and defensive rationalizations. It develops over time and contextual factors influence its expression. The nature of a smoker’s identity can play an important role in smoking cessation.

Stopping smoking, especially before middle age, has enormous health benefits because it reduces the risks of mortality and morbidity caused by smoking-attributable diseases (Doll, Peto, Boreham, & Sutherland, 2004; Dresler, León, Straif, Baan, & Secretan, 2006; Pirie et al., 2013). If people quit smoking when young, they not only have a huge improvement in their life expectancy, but they are also more likely to have a better quality of life (Jia, Zack, Thompson, & Dube, 2013) and improved mental health (Taylor et al., 2014) for the rest of their lives. Moreover, given that people commonly start childbearing between their early twenties and midthirties, quitting while young is very important in terms of avoiding future risks of maternal and paternal smoking on pregnancy and children’s health. However, as 75% of ever smokers in England do not manage to quit by then (West & Brown, 2012), there is a need to improve smoking cessation efforts in younger people. This will require a greater understanding of influences and motivations in relation to their smoking and smoking cessation behaviors. Identity has been proposed as an important motivational force influencing health behaviors (Kearney & O’Sullivan, 2003; Oyserman, Fryberg, & Yoder, 2007; West, 2006), but evidence on the nature of young adults’ identities relating to smoking and how identity might underpin smoking and smoking cessation is scant.

Identity has been studied from different theoretical traditions within a variety of disciplines, including psychology, sociology, philosophy, and linguistics; these approaches differ markedly in how they construe identity, including its definition, how it is developed, and the processes through which it operates (Vignoles, Schwartz, & Luyckx, 2011). An integrative perspective proposes that identity simultaneously has personal, relational, and collective contents and that coexisting identity aspects are established, maintained, and revised through conscious or automatic processes depending on the intra-/interpersonal and sociocultural contexts throughout the life span (Vignoles et al., 2011). Taking the implications of this comprehensive perspective as the starting point for our conceptual view on identity, we consider it as a psychological construct that comprises people’s mental representations of themselves, including their thoughts (categorized into self-labels, attributes, and personal rules) and images about themselves and the feelings they attach to these (West, 2006).

## Identity in Theories of Motivation and Behavior Change

Identity has been recognized as driving behavior, and theories of motivation and behavior change have suggested different ways through which this influence might be operating. Identity motives (tendencies toward or away from certain identities): the motive for self-esteem, continuity, distinctiveness, meaning, efficacy, and belonging, have been proposed as energizing identity definition and enactment, and providing the basis for any identity processes to potentially drive behavior (Vignoles, 2011).

Social identity theory (Tajfel & Turner, 1986) suggests that people identify themselves with the social groups to which they belong; evaluate the meanings, beliefs, and feelings they attach to their group memberships; and are motivated to act in ways that maintain a positive social identity. Building on these premises, identity-based motivation theory (Oyserman, 2007, 2009; Oyserman et al., 2007) further argues that people are motivated to engage in identity-congruent behaviors regardless of the costs or benefits of the behavior. Therefore, current or possible identities (aspirational or feared future identities) triggered by the context in any given moment will influence individuals’ action readiness (self-control to engage in identity-congruent behavior) and procedural readiness (making sense of the world by using identity-congruent cognitions). The bidirectional relationship between identity change and behavior change is emphasized in identity change theory (Kearney & O’Sullivan, 2003), which posits that a conflict between values/goals and behavior initiates a step toward behavior change, and if successful, this behavior change can lead to an identity shift that will further strengthen the new behavior. The PRIME theory, in which PRIME stands for ‘Plans’, ‘Responses’, ‘Impulses’, ‘Motives’ and ‘Evaluations’, (West, 2006) adds to these debates by placing identity within a comprehensive and hierarchically structured model of the motivational influences on behavior, including automatic responses and impulses, motives, and self-conscious evaluations and plans. A key tenet of PRIME theory is that identity has an important role in controlling behavior by generating potentially strong wants and needs against competing desires and impulses arising from the immediate situation. For example, a personal rule arising from attachment to an identity as someone who is slim can override a strong biological drive to eat.

## Quantitative Evidence Regarding Smoker Identity

Quantitative studies, which were considerably heterogeneous in terms of the ways in which they assessed smoker identity, have reported that smokers do not necessarily internalize a smoker identity, instead they deny being a smoker (Berg et al., 2009; Choi, Choi, & Rifon, 2010; Leas, Zablocki, Edland, & Al-Delaimy, 2014; Levinson et al., 2007; Ridner, Walker, Hart, & Myers, 2010) or identify themselves with alternative self-labels, such as “social smoker” (Song & Ling, 2011). Those with a smoker or social smoker identity are less likely to intend to quit smoking (Falomir & Invernizzi, 1999; Høie, Moan, & Rise, 2010; Moan & Rise, 2005; Song & Ling, 2011), more likely to increase the frequency of smoking (Hertel, Mermelstein, & Robin, 2012), and more likely to respond defensively to persuasive antitobacco messages (Freeman, Hennessy, & Marzullo, 2001). Having positive feelings about one’s identity as a smoker has been reported to undermine efforts to stop smoking (Tombor, Shahab, Brown, & West, 2013). In addition, those with greater identity and attitudinal conflicts about quitting have been found to prefer gradual cessation to abrupt cessation (Wee, Shahab, Bulgiba, & West, 2011). There is also evidence that people’s self-concepts relating to smoking change during behavioral support for smoking cessation (Shadel, Mermelstein, & Borrelli, 1996), and strengthening ex-smoker identity is associated with self-reported and carbon monoxide verified 4-week abstinence (West, Walia, Hyder, Shahab, & Michie, 2010).

## Aims and Scope of the Meta-Ethnography

Qualitative studies can contribute greatly to our understanding of smoker identity by offering potentially deep insights into the meanings young adults attach to their smoker identities and how changes in these could shape their smoking and smoking cessation behavior. We therefore undertook a systematic literature review of the available qualitative evidence on this topic, using the meta-ethnographic method of synthesis to provide new insights (Noblit & Hare, 1988). This was achieved by translating key concepts across studies and developing a single comprehensive framework, so extending understanding beyond that offered via the individual qualitative studies (Noblit & Hare, 1988). Meta-ethnography has been deemed an effective method of synthesizing qualitative findings in a systematic way (Campbell et al., 2011) and has already been widely used to advance knowledge across the medical and social sciences (Hubbard, McLachlan, Forbat, & Munday, 2012; Malpass et al., 2009; Ring et al., 2011; Smith, Pope, & Botha, 2005). While we recognize that smoker identity is but one of several identities young adults explore during the emergence of adulthood (Arnett, 2000), this paper focuses on smoker identity alone due to the pragmatic needs of meta-ethnographies and for theoretical coherence. The present meta-ethnography addressed the following questions:

1 How do smokers perceive their smoker identity, and what factors shape their beliefs, meanings, and attitudes attached to it?
2 How does identity change preceding or following smoking cessation?
3 How does smoker identity influence smoking and smoking cessation?

## Methods

### Search Strategy

Electronic databases (PubMed/MEDLINE, PsychInfo, Web of Knowledge, and CINAHL [Cumulative Index to Nursing & Allied Health] Plus) were searched from the inception of each database on September 19, 2013, by using the terms: (identit* OR self-concept OR self-identit* OR self-labe*) AND (smoker OR smoking OR tobacco use) AND (adolescen* OR youn* OR teenage* OR studen* OR pregnan*). Searches for MeSH (Medical Subject Headings) terms were added where applicable to the database. The search was restricted to journal articles published in English, and gray literature was excluded. Records were downloaded and duplicates removed. To identify further relevant articles, corresponding authors of the included papers were contacted, and reference lists of the included papers were hand-searched.

### Exclusion and Inclusion Criteria

To define inclusion criteria for papers in the meta-ethnography, we used the PICo (Population, phenomena of Interest, Context) framework for qualitative studies, which is an adaptation of the widely used PICOTS (Population, Intervention, Comparison, Outcome, Time-frame, Study design) framework (The Joanna Briggs Institute, 2011). According to PICo, papers were eligible for inclusion if: (a) the study population comprised young adults (age 16–34) who were current smokers or ex-smokers at the time of enrollment; (b) the phenomenon of interest in the paper was “identity” defined as participants’ thoughts, images, and/or feelings about themselves; (c) the context in which the phenomenon was considered was cigarette smoking; and (d) the papers were original, qualitative research articles that were published in peer-reviewed journals and written in English. Reviews, book chapters, dissertations, commentaries, and protocol papers were excluded. Two reviewers, Ildiko Tombor and Aleksandra Herbec, independently screened the papers against the inclusion criteria starting with titles and abstracts (*n* = 1,694) and then moving on to the full-text screening of the papers (*n* = 123). After all titles and abstracts were screened, there was an acceptable initial interrater agreement (Cohen’s κ = 0.61) between the two reviewers. Discrepancies were discussed and, if needed, resolved by Lion Shahab. From all full-text articles screened, 17 met the inclusion criteria.

### Quality Assessment

The quality of the 17 included papers was assessed by Ildiko Tombor and Aleksandra Herbec independently using the National Institute for Health and Clinical Excellence (NICE) quality appraisal checklist for qualitative studies (National Institute for Health and Clinical Excellence, 2009). Depending on the number of criteria fulfilled (minimum = 0; maximum = 14), papers were categorized into: high (11–14), medium (7–10), or low (0–6) quality, with 82.4% initial agreement between the two researchers. Disagreements were resolved through discussion. The overall quality of the papers was found to be good: eight were categorized as high quality, eight as medium quality, and one as low quality. Common weaknesses, as judged by the quality appraisal checklist, were: lack of triangulation, inadequate description of the role of the researcher, how the researcher may have influenced the results, and the context of the study. Given the lack of consensus in the literature about the quality appraisal of qualitative papers (Campbell et al., 2011; Dixon-Woods, Fitzpatrick, & Roberts, 2001; Toye et al., 2013), we did not exclude the low-quality paper.

### Data Extraction

Data on study characteristics (aims, design, setting, target population, and sample size) and participants’ characteristics (age, gender, ethnicity, and smoking status) were extracted by Ildiko Tombor and verified by Aleksandra Herbec. Ildiko Tombor also extracted key concepts relating to smoker identity, with dual data extraction conducted on 50% of eligible studies by Aleksandra Herbec. Inconsistencies were resolved through discussion.

### Data Analysis

We conducted the analysis by following the principles of meta-ethnography (Noblit & Hare, 1988). First, all studies were read to identify relevant key concepts. The extracted key concepts, in which the meaning of the original text was preserved, were then compared with each other and synthesized to generate a list of first-order interpretations. Overarching second-order interpretations were then derived from first-order interpretations expressing similar concepts. The final level of synthesis involved the development of a comprehensive framework by establishing the relationship between second-order interpretations to form third-order interpretations. The analysis involved repeated discussions between members of the research team and several iterations of the framework until agreement was reached.

## Results

**Figure 1 fig1:**
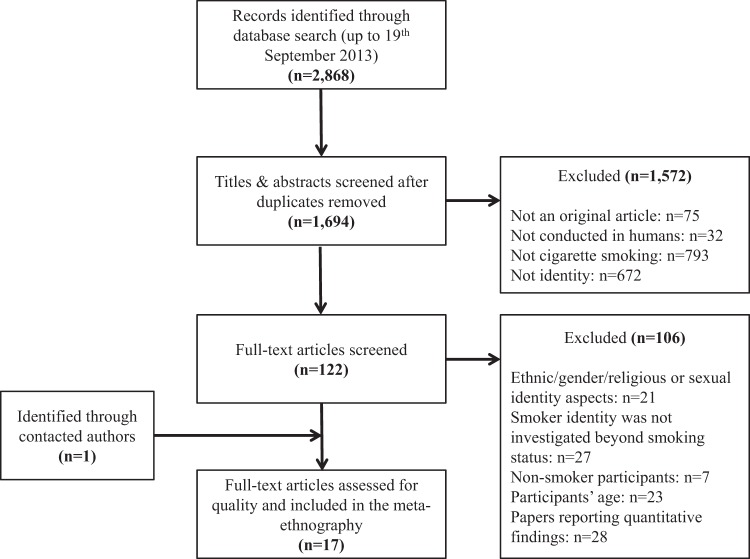
Flow chart for paper selection.

Figure 1 shows the paper selection process. Seventeen qualitative papers reporting on 14 studies, collectively including about 500 smokers from Western industrialized countries, met our inclusion criteria. Multiple papers with the same participants were included, since each paper contributed different findings that broadened understanding of the study data. The study characteristics of included papers and the list of excluded papers after full-text screening with reasons for exclusion are reported in the supplements (Table S1 and Table S2, respectively).[Fig-anchor fig1]

In total, 248 key concepts were identified and synthesized into 143 first-order interpretations (Table S3). The most frequent first-order interpretation was “identification with a social/casual smoker identity rather than a smoker identity.” First-order interpretations were synthesized into 15 second-order interpretations and subsequently four third-order interpretations: (a) contributory factors to identity, (b) identity in relation to smoking, (c) contextual and temporal patterning, and (d) behavior in relation to smoking (see Table 1). Third-order interpretations with their contributing second-order interpretations are discussed in detail below.[Table-anchor tbl1]

### I. Contributory Factors to Smoker Identity (Individual, Social, and Behavioral Factors)

#### 1. Being a smoker serves other personal identity functions

Internalization of a smoker identity was found to be frequently grounded in individuals’ desire to establish aspirational identities, including being a mature, sophisticated, glamorous, or self-confident person.

I think when you see those pictures in New Weekly, and there’s, like, Elle Macpherson, and she’s got a cigarette in her hand, I think it’s pretty obvious what people think, because they look up to them as iconic, then they think, well they can do it, I can do it. (Gilbert, 2007, p. 7)

Smoking tended to be seen as an identity trademark that people could use to distinguish themselves from others and to express their individuality, other identity aspects (e.g., identity as a young person) and personal attributes (e.g., being a fun-loving person who prioritizes pleasure over health awareness; Gilbert, 2007; Lennon, Gallois, Owen, & McDermott, 2005; MacFadyen, Amos, Hastings, & Parkes, 2003; Moffat & Johnson, 2001; Rooke, Amos, Highet, & Hargreaves, 2013; Scheffels, 2008, 2009; Scheffels & Schou, 2007; Wiltshire, Amos, Haw, & McNeill, 2005).

I play the guitar and when I go with my friends from the band to these music bars, smoking kind of becomes part of that rock n’ roll image. (Scheffels, 2008, p. 120)

#### 2. Being a smoker has social benefits

It was found that being a smoker could enhance social power, since it helped individuals to feel included, express membership of their social groups, and maintain an identity that was valued within these groups. However, smoking was not necessarily experienced as pleasurable; rather, engaging in smoking was essential for social acceptance: the benefits of being part of a group of smokers and establishing a smoker identity appeared to outweigh the risks of smoking (Brown, Carpenter, & Sutfin, 2011; Gilbert, 2007; Hoek, Hoek-Sims, & Gendall, 2013; Hoek, Maubach, Stevenson, Gendall, & Edwards, 2013; Johnson et al., 2003; Lennon et al., 2005; MacFadyen et al., 2003; Scheffels, 2008, 2009; Scheffels & Schou, 2007; Wiltshire et al., 2005).

Like I know it’s bad for me but I can just do it and fit in or I can say no and run the risk of being out-casted or something like that. (Hoek, Maubach et al., 2013, p. 263)

#### 3. Social environment influences the enactment of a smoker identity

Studies found that being a smoker could be considered an acceptable, even desirable, identity around friends and other smokers. However, as the context changes to either a more private or a professional situation (e.g., being around family members, employers, or clients), the smoker identity often needed to be hidden.

A really big difference is in the type of image that you give off if you’re walking across campus smoking than if you’re in a party smoking. (Brown et al., 2011, p. 1202)

While there was evidence that some smokers might strengthen their smoker identification as a counter to the stigma associated with smoking (Brown et al., 2011; Hoek, Hoek-Sims et al., 2013; Hoek, Maubach et al., 2013; Johnson et al., 2003; Rooke et al., 2013; Scheffels, 2008, 2009; Wiltshire et al., 2005), awareness of the negative social discourse associated with smoking frequently resulted in a denial of being a smoker, even in front of strangers, in order to avoid destroying one’s reputation and to make a good first impression (cf. II. below).

I don’t smoke at work cause I work in a posh, posh shop and don’t want clients thinking “oh there’s another wee girl smoking, it looks so silly they think they’re so grown up.” (Wiltshire et al., 2005, p. 610)

#### 4. Other behaviors influence smoker identity

Being in situations where alcohol was being consumed was repeatedly identified as an important factor overriding personal nonsmoking rules, and it appeared to make smoking more acceptable and reduced cognitive dissonance. Additionally, being a “nonsmoker who smokes when drinking” could become part of a smoker’s self-definition; thus, maintaining a dual identity as smoker and nonsmoker and ultimately reducing identity conflicts.

Well I hate it. [the person strongly identified himself as a nonsmoker] Like if someone lit one up right now I’d probably vomit. It just makes me feel so sick. It’s weird, I just yeah, well, after I’ve had a drink I just don’t care. (Hoek, Maubach et al., 2013, p. 264)

The ban on smoking in indoor spaces prompted smokers to pay closer attention to how they presented their identities when returning to indoor drinking locations after smoking outdoors. Nonetheless, a sociable smoker identity could be maintained with the introduction of beer gardens and other forms of pleasurable outdoor spaces where smoking is permitted (Brown et al., 2011; Hoek, Maubach et al., 2013; Johnson et al., 2003; Lennon et al., 2005; Rooke et al., 2013; Wiltshire et al., 2005).

When you are outside talking to people, that’s when you meet new people because they are, there are areas where people smoke is really social [. . .] you go outside and everyone automatically starts talking to each other and it’s very social. (Rooke et al., 2013, p. 113)

### II. Identity in Relation to Smoking

#### 5. Smoker identity is multifaceted

The research found that multiple smoker identities appeared to coexist with one salient smoker identity at any given moment. The list of different smoker identities that we identified is reported in the supplements (Table S4).

I don’t see myself as a smoker but I see myself as a social smoker. It’s almost like they’re almost mutually exclusive. (Hoek, Maubach et al., 2013, p. 263)

Smoker identities could be characterized by different dimensions: whether individuals perceived themselves as having an active or a passive role in the development of a given smoker identity, whether they saw the health effects of smoking as personally relevant to them and whether they thought about smoking as an ongoing behavior (Berg et al., 2010; Brown et al., 2011; Hoek, Maubach et al., 2013; Johnson et al., 2003; Lennon et al., 2005; MacFadyen et al., 2003; Moffat & Johnson, 2001; Rooke et al., 2013; Scheffels, 2008, 2009; Wiltshire et al., 2005).

[Smoking has] very negative health effects, but I don’t feel like those really affect me as much [because I am] an occasional smoker. (Brown et al., 2011, p. 1201)

#### 6. A nonsmoker identity is confirmed by defensive rationalizations

Individuals often used forms of defensive rationalization to justify to themselves that they were not smokers, despite smoking cigarettes. Such rationalizations focused on the specifics of smoking behaviors, such as they smoked less and less often than others around them, or they would never buy cigarettes for themselves, never smoke alone, and never deeply inhale tobacco smoke.

If I’m not buying them, I’m not a smoker. If I’m only getting them off people, then it’s not an issue. Because I’m not wasting my money. (Hoek, Maubach et al., 2013, p. 263)

Smokers reported that they felt in control of their smoking and discussed ease of quitting, even though they might have never tried. Those who denied being a smoker preferred a social smoker self-label, consistently stated that they were not addicted to cigarettes, and attributed any cravings to the social aspects of smoking (Amos, Wiltshire, Haw, & McNeill, 2005; Berg et al., 2010; Brown et al., 2011; Hoek, Maubach et al., 2013; Johnson et al., 2003; MacFadyen et al., 2003; Moffat & Johnson, 2001; Rooke et al., 2013; Scheffels, 2009; Scheffels & Schou, 2007; Wiltshire et al., 2005).

I could stop just like that. I don’t smoke a lot so I would find it easy to quit [daily smoker who did not consider herself a smoker and never tried to quit]. (Amos et al., 2005, p. 184)

#### 7. Negative images are associated with being a smoker

Individuals generally associated smoking with negative characteristics relating to physical appearance (e.g., having yellow teeth and smelling), psychological characteristics (e.g., being desperate and anxious), and future aspirations (e.g., not being successful).

I wish I never smoked. It costs money; it’s disgusting; it’s dirty; it makes your clothes smell bad; it makes your hair smell bad; it makes your teeth ugly; and it just sucks your whole life into it. (Johnson et al., 2003, p. 394)

They also recognized that others might portray them similarly, which evoked feelings of shame and regret. These stereotypical negative smoker characteristics were seen as incompatible with individuals’ own self-image; thus smokers wanted to avoid this unattractive identity either by not being seen publicly as a smoker or by stopping smoking (Berg et al., 2010; Brown et al., 2011; Hoek, Hoek-Sims et al., 2013; Hoek, Maubach et al., 2013; Johnson et al., 2003; MacFadyen et al., 2003; Rooke et al., 2013; Scheffels, 2008, 2009).

The label that we get is [. . .] “oh, you’re smoking,” you know, brush away; it’s not a big deal to them. But some people, it’s just, you know—just say it’s kind of like being a teenager: you get the turned-up noses and the bad looks from people because you are a smoker, and, you know, I wish I wasn’t a smoker because I’d give those faces to people. (Johnson et al., 2003, p. 391)

#### 8. Negative images are associated with becoming an addicted smoker

Addicted smokers were often referred to as “real smokers” and were portrayed negatively (e.g., they had lost control of smoking and had urges to smoke) by nondaily smokers. Participants, regardless of whether they were daily or nondaily smokers, reported controlling when and how many cigarettes they smoked thereby enabling them to avoid addiction and being looked down on. Fear of becoming addicted appeared to underlie individuals’ intentions of dissociating themselves from an addicted smoker self-image and expressing an identity as a “smoker in control” (Amos et al., 2005; Brown et al., 2011; Hoek, Maubach et al., 2013; Johnson et al., 2003; MacFadyen et al., 2003; Moffat & Johnson, 2001; Scheffels, 2008, 2009; Scheffels & Schou, 2007).

I don’t want to become addicted to where I have to smoke many a day [. . .] that’s the only reason I don’t smoke as much. (Brown et al., 2011, p. 1201)

#### 9. Being a smoker causes identity conflicts

There were evident discrepancies between having a smoker identity and other current or future identity aspirations, such as being an athlete or a successful person. Likewise, being accepted by an immediate social group of smokers and simultaneously stigmatized by society more generally appeared to create fundamental internal conflicts. Such tension could then trigger a shift between smoker identities, including a complete denial of being a smoker (Brown et al., 2011; Hoek, Maubach et al., 2013; Johnson et al., 2003; Moffat & Johnson, 2001; Rooke et al., 2013).

I done work experience a while back and I didn’t want him to know I was smoking [. . .] I don’t know, it’s not something you are proud of, the fact that you smoke. Even though I do enjoy it. (Rooke et al., 2013, p. 111)

#### 10. Being a smoker is a positive identity

Studies found that there were smokers with various life-circumstances for whom being a smoker was still a positive identity as they considered their internalized smoker identity to be a relatively better life choice and morally superior to being a drug user or alcoholic. Similarly, being a light smoker or nondaily smoker could be deemed superior to being a “pack-a-day” smoker or addicted daily smoker. Comparing themselves favorably with others in their immediate social environment thus enabled smokers to retain a relatively positive identity despite the perceived negative connotations of smoking (Brown et al., 2011; Hoek, Maubach et al., 2013; Johnson et al., 2003; Lawson, 1994; Scheffels, 2009).

I’m better than other girls in the projects who are running around with different boys, selling their bodies for drugs, and writing cold checks ’cause I just smoke. (Lawson, 1994, p. 68)

### III. Contextual and Temporal Patterning

#### 11. Development of a smoker identity is an evolutionary process

The term “becoming a smoker” was used to describe individuals’ experiences of internalizing a smoker identity. A smoker identity was adopted over time but, once established, seldom consciously questioned. Initiation of a smoker identity was commonly triggered by an unsuccessful quit attempt, buying cigarettes for oneself, and the recognition that smoking was no longer limited to particular times or places (Amos et al., 2005; Berg et al., 2010; Johnson et al., 2003; Kishchuk, Tremblay, Lapierre, Heneman, & O’Loughlin, 2004; Moffat & Johnson, 2001; Scheffels, 2008, 2009; Wiltshire et al., 2005).

I never used to think of myself as a smoker, it was just as someone who smoked. One day I couldn’t be bothered smoking and I wanted to stop but I couldn’t stop. And I didn’t even know I was addicted. (Wiltshire et al., 2005, p. 608)

#### 12. Possibility for a shift between smoker identities

Individuals were found to be purposefully shifting between their smoker identities and the possibility of rapidly transitioning between these identities enabled smokers to act in different ways in different contexts (e.g., in front of friends vs. employers).

Say you have a big wide wardrobe, and you have certain clothes for certain things. And say you’ll have a certain outfit that you’ll wear to school, and you have a certain outfit that you will wear to, say, the bar. The cigarette would be the outfit I would wear to the bar, but it’s not the outfit I would wear to school. (Johnson et al., 2003, p. 393)

As a result, concurrent smoker identities did not necessarily cause identity conflicts or tension. Long-term changes in smoker identities from being a smoker to a nonsmoker could, however, provide individuals with a good basis for maintaining smoking cessation (cf. IV. below; Johnson et al., 2003; Scheffels, 2008, 2009).

#### 13. Future smoker identities exist

Complementing their current smoker identities, smokers proffered mental representations of themselves as they might be in the future. They suggested future personal situations, in which smoking would not be an option (e.g., they would not smoke if they had a child).

When I see my future I don’t see myself as a smoker, I don’t see myself even as a social smoker. (Hoek, Maubach et al., 2013, p. 263)

On the other hand, those who recognized that smoking was an addiction for them or who had already tried to quit smoking but failed tended not to hold future nonsmoker identities. Similarly, individuals who associated being a smoker with other positive identity aspects, such as being an adult, were reluctant to imagine themselves other than as a smoker in the future (Amos et al., 2005; Brown et al., 2011; Hoek, Maubach et al., 2013; Johnson et al., 2003).

### IV. Behavior in Relation to Smoking

#### 14. Smoker identities influence making a quit attempt

Smoker identities appeared to influence individuals’ intentions to make a quit attempt. The conflict between smoker identities and other valued identity aspects (e.g., being a good mother) could generate motives to attempt cessation. Conversely, factors that could undermine a desire to quit smoking included having positive feelings about being a smoker, incorporating positive risk acceptance into one’s smoker identity, or internalizing a nonsmoker identity despite smoking cigarettes. In addition, those with an established smoker identity formulated only vague plans to stop smoking in the future (Amos et al., 2005; Berg et al., 2010; Brown et al., 2011; Hoek, Maubach et al., 2013; Johnson et al., 2003; Kishchuk et al., 2004; Lennon et al., 2005; MacFadyen et al., 2003; Scheffels, 2009; Scheffels & Schou, 2007).

I don’t really think about myself as a smoker, so it is hard to think about when you would quit. (MacFadyen et al., 2003, p. 495)

#### 15. Smoker identities influence long-term abstinence

Having a strong belief about being able to become a nonsmoker, and formulating plans to leave a smoker self behind could help individuals to abstain from smoking. Being someone who managed to quit smoking and did not want to smoke ever again could also give strength and feelings of positive self-evaluation (Johnson et al., 2003). Despite this, where smoking cessation was induced by the social environment and was not grounded in an individual’s own wants and needs to remain abstinent, the possibility of future smoking remained. Similarly, lack of a firm nonsmoker identity appeared to make individuals vulnerable to start smoking again.

You never know, one day something will click and I will smoke. (Johnson et al., 2003, p. 392)

### Framework for the Synthesis

**Figure 2 fig2:**
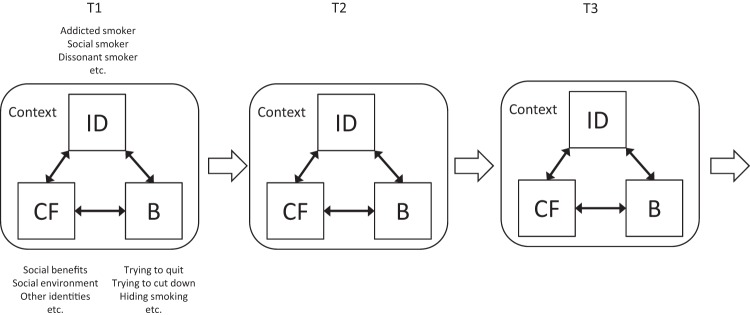
Framework for the interpretation of smoker identity and identity change in young adults.

Figure 2 shows the framework for our synthesis. In this framework, context (e.g., being with one’s family at home, being with one’s friends, etc.) conditions the current identity and the factors that contribute to it. Thus, identities may be different and evolve differently in different contexts. In any given context, interrelated individual (e.g., desire to establish aspirational identities through being a smoker), social (e.g., enacting an identity that is appropriate to the relevant social environment), and behavioral factors (e.g., altering personal nonsmoking rules if consuming alcohol) contribute to a multifaceted smoker identity that incorporates thoughts (e.g., defensive rationalization), images (e.g., negative self-image as a smoker), and feelings (e.g., identity conflicts due to being a smoker). Therefore, smoker identities are perceived as important in people’s self-definition if they satisfy identity motives (Vignoles, 2011), including the need to maintain/enhance self-esteem, distinctiveness from others (e.g., nonsmokers or daily smokers), and feelings of belonging. On the other hand, if being a smoker conflicts with motives for self-esteem and/or belonging, young adults hide their smoker identities and use defensive psychological mechanisms to protect their positive self-esteem and social identity. Moreover, salient smoker identities triggered by particular contexts can evoke identity congruent cognitions and influence young adults’ self-regulation, such that smoking could be acceptable if consuming alcohol or unacceptable if not deemed the kind of behavior practiced by other in-group members.[Fig-anchor fig2]

A key aspect of the contextual patterning of smoker identity is the possibility for rapid shifts between identities, which provide the basis either for the elimination of internal conflicts or for the behavior change. Nevertheless, firmly established smoker identities might increase/decrease the importance of the influence of the contributory factors in a reciprocal association, because if having, for example, a confirmed smoker identity that neither conflicts with the person’s identity motives nor causes internal conflicts between other identity aspects, then it will decrease the importance of the social context in terms of inducing change in behavior or shifts between smoker identities.

The temporal patterning of smoker identity involves long-term evolutionary processes. In line with theories of social comparison (Festinger, 1954), social identity (Tajfel & Turner, 1986), and identity-based motivation (Oyserman, 2009), a strong social embedment is evident not just in terms of establishing smoker identities, but also in the context of managing these identities to maintain a positive social identity. Therefore, once a socially acceptable smoker identity is established (e.g., being a nondaily smoker), people can use it as a means of self-enhancement by comparing themselves to others who are perceived similar to themselves in many aspects (e.g., similar age), but who are also worse off in terms of smoking-related characteristics (e.g., being and addicted daily smoker). In addition, smoker identities can be drivers for health promoting or health destructive behaviors (e.g., the identification with a nonsmoker identity that is grounded in defensive rationalization could deter smokers from making quit attempts and therefore undermine any efforts to achieve long-term abstinence). Finally, individuals’ smoking and cessation behaviors can also facilitate the establishment of new smoker identities over time (e.g., an unsuccessful quit attempt could induce a confirmed smoker identity).

## Discussion

The smoker identity phenomenon was considerably more complex than a binary construct (i.e., being a smoker vs. nonsmoker), and many current smoker identities were identified. Moreover, as people proffered mental representations of themselves regarding who they would like to become (e.g., nonsmoker identity) and who they are afraid of becoming (e.g., identity as an addicted smoker) in the future, our findings suggest that identity aspects related to health behaviors can complement people’s possible selves (Markus & Nurius, 1986). Young adults could hold different smoker identities concurrently, and individual, social, and behavioral factors could all shape how they perceived their smoker identities and the attitudes they attached to these. Smoker identities might not be established automatically and in tandem with smoking initiation but may be adopted over time and changed intentionally depending on the context. Additionally, different smoker identities could both facilitate and inhibit smoking cessation.

This meta-ethnography highlights the potential for long-term changes and short-term fluctuations in young adults’ smoker identities that seem to be driven by increasing discrepancies between aspirational and current identities (Kearney & O’Sullivan, 2003) and the desire to maintain a positive social identity in the momentary context (Tajfel & Turner, 1986). This could be particularly relevant during young adulthood when most people are in the early stages of their professional careers, because although the sense of belonging to social groups are still highly valued, discrepancies can occur between these social identities and newly established or aspirational identities related to their professional roles. Our findings support previous studies that have shown that many smokers deny their smoker identities (Berg et al., 2009; Choi et al., 2010; Leas et al., 2014; Levinson et al., 2007; Ridner et al., 2010). Consistent with quantitative findings (Falomir & Invernizzi, 1999; Høie et al., 2010; Moan & Rise, 2005; Tombor et al., 2013) and theories that suggest a dynamic between identity change and behavior change (Kearney & O’Sullivan, 2003; West, 2006), we found that the extent to which smokers internalize different smoker identities provides a potentially important subjective determinant of motivation to engage in or refrain from smoking.

The synthesis suggests that identity processes, including the internalization/revision of different identities with underlying identity motives and contextual influences, matter in smoking cessation. Therefore, interventions should take account of young adults’ different smoker identities, including those who might have a nonsmoker identity despite smoking. Moreover, in order to better personalize behavioral support for cessation, smoker identities should be considered together with other identity aspects. Smokers should be encouraged to articulate what their smoker identities mean to them in order to explore underlying identity motives (Vignoles, 2011). Building on the identity motives identified, interventions should help smokers work out how their identity could be complete without smoking. In particular, interventions should facilitate the establishment of an identity that would satisfy the need to increase self-esteem, increase distinctiveness from others, and maintain feelings of belongings without smoking. Further, potentially important targets for interventions to promote identity change and behavior change could be tackled by established behavior change techniques (Michie et al., 2013). These include the monitoring of people’s commitment to a nonsmoker identity throughout the smoking cessation process, and strengthening it if needed; drawing smokers’ attention to the discrepancies between their smoker identities and other aspirational identities; and identifying positive role model(s) together with smokers to strengthen their desire to change identity. Finally, interventions should emphasize that despite being addicted to cigarettes or finding it difficult to quit, it is always possible to change one’s identity and become a nonsmoker, which could eventually reinforce sustained behavior change.

One limitation of this meta-ethnography is that only journal articles published in English were included, and all reported studies were conducted in Western industrialized countries. Moreover, even though our search strategy led us to identify a broad range of studies covering a variety of topics in electronic databases and we contacted authors of included papers to identify further journal articles, gray literature searching was not carried out. Relevant findings might therefore have been omitted.

Future studies should explore smoker identities and how these influence behavior in different age groups and cultural contexts. We need to advance our knowledge of the ways in which identity motives underlying a smoker identity can be explored and techniques to help people establish an identity that fulfils these needs without smoking. A better understanding of situations with a potential to trigger long-term changes in smoker identities and the ways that smoker identities might interact with other health-related identity aspects (e.g., physical activity, alcohol consumption) would also be needed.

To our knowledge, this meta-ethnography is the first to provide an overview of smoker identity and its potential role in smoking and smoking cessation in young adults. A systematic synthesis of qualitative evidence emphasized the complex nature of smoker identities; the interaction with individual, social, and behavioral factors; the possibility of change; and the potential of different smoker identities in influencing people’s smoking and smoking cessation. Our findings reinforce the view that smoking cessation interventions should address people’s identities relating to smoking and facilitate the establishment of a firm identity in which there is no place for smoking.

## Supplementary Material

10.1037/hea0000191.supp

## Figures and Tables

**Table 1 tbl1:** Formulation of First, Second, and Third-Order Interpretations

Examples of key concepts identified in original research articles	Examples of first-order interpretations (ID numbers of all related first-order interpretations as reported in Table S3)	Second-order interpretations	Third-order interpretations
“I think smokers seem so relaxed . . . and I want to look relaxed.” (Scheffels & Schou, 2007, p. 171)	Smoking to achieve an ideal self (19, 23, 25, 37, 38, 39, 41, 42, 43, 48, 61, 99, 102, 103, 121, 134)	1. Being a smoker serves other personal identity functions	I. Contributory factors to identity (individual, social, and behavioral factors)
“Wasn’t that interested in it [smoking during school years]. I was just into my PE [Physical Education]. But now that’s all gone [desirable social status]. I still go for my run, still go to the gym, but with bricklaying everybody else smokes” (Wiltshire et al., 2005, p. 611)	Smoking to express an identity, which is in accordance with what is valued by the social group to maintain social status (40, 44, 51, 59, 62, 67, 101, 104, 105, 137, 140, 141)	2. Being a smoker has social benefits	
“If a complete stranger was to come up to me and ask if I smoked, I would say ‘no,’ just in case they are looking for someone for a job.” (Wiltshire et al., 2005, p. 611)	Being a smoker is cool with other smokers, but not acceptable in a professional environment (22, 34, 35, 36, 56, 136, 138)	3. Social environment influences the enactment of a smoker identity	
“[not a smoker] because I don’t smoke, right? I don’t smoke unless I’m drinking.” (Johnson et al., 2003, p. 391)	Consuming alcohol liberates smokers from their nonsmoker identity and reconcile dissonance that would normally keep them away from smoking (20, 34, 52, 57, 58, 117, 132, 133)	4. Other behaviors influence smoker identity	
“I define myself as an occasional smoker because I don’t really like it, I don’t even like the taste of it. I do it whenever I’m under stress because it helps me to organize my thoughts and be alone.” (Berg et al., 2010, p. 966)	Identification with a nondaily/occasional smoker identity as opposed to a smoker identity (31, 54, 63, 64, 65, 66, 68, 70, 71, 72, 74, 78, 82, 84, 85, 86, 89, 108, 116, 122, 123, 129)	5. Smoker identity is multifaceted	II. Identity in relation to smoking
“Occasionally I smoke, but I wouldn’t say I’m a smoker; it’s like saying that occasionally I drink versus saying I’m an alcoholic.” (Brown et al., 2011, p. 1201)	Identifying oneself as a smoker depends on whether the person thinks that he/she is addicted versus smokes due to habit (1, 2, 3, 16, 18, 21, 24, 26, 28, 45, 47, 60, 96, 106, 110, 111, 112, 113, 120)	6. A “nonsmoker” identity is confirmed by defensive rationalization	
“It’s not cool smelling like a cigarette all the time and people who don’t smoke smell you, they are like, yeah, I don’t know, give you a look or something . . . it’s pretty shaming.” (Hoek, Hoek-Sims et al., 2013, p. 614)	Do not want to be seen as a smoker due to feelings of guilt and shame associated with it (27, 29, 49, 75, 130, 131, 135, 139, 142)	7. Negative images are associated with being a smoker	
“How can I be addicted if I don’t even buy cigarettes, I just get one from a friend once in a while.” (Moffat & Johnson, 2001, p. 672)	Rejection of being addicted to maintain identity as a person who keeps smoking under control (5, 6, 7, 8, 9, 10, 31, 32, 107, 109)	8. Negative images are associated with becoming an addicted smoker	
“Um, it makes it all, you think why did I do this, you know, I don’t need to do this, this is ridiculous. But then I just do it again anyway. I dunno. It’s ridiculous, it’s absolutely ridiculous.” (Hoek, Maubach et al., 2013, p. 264)	Internal conflicts due to perceived superior status as a nonsmoker but engaging with stigmatized behaviour (30, 46, 49, 50, 53, 55, 80, 100, 136)	9. Being a smoker causes identity conflicts	
“I’m proud to be just a smoker ‘cause my parents are alcoholics. My dad is drinking himself to death at age 36. My mother drinks beer every day. I only smoke one cigarette three times a day.” (Lawson, 1994, p. 69)	Being a smoker gives feelings of superiority above other substance users (90, 91, 92, 107)	10. Being a smoker is a positive identity	
“‘I smoke’ rather than ‘I am a smoker.’” (Kischuk et al., 2004, p. 496)	Smoking is not part of identity	11. Development of a smoker identity is an evolutionary process	III. Contextual and temporal patterning
“I don’t socially smoke any more, I buy my own, I think that’s always a sign when you’re a proper smoker, you stop borrowing off other people.” (Witshire et al., 2005, p. 608)	Identification with a smoker identity is associated with buying one’s own cigarette		
“I’m a regular smoker, not exactly heavy. I’m not a pack-a-day [smoker] yet.” (Johnson et al., 2003, p. 394)	Acceptance of a “smoker identity” is unquestioned, and it is adopted by time (13, 74, 88, 95, 97, 98, 115, 118, 119)		
“Say you have a big wide wardrobe, and you have certain clothes for certain things. And say you’ll have a certain outfit that you’ll wear to school, and you have a certain outfit that you will wear to, say, the bar. The cigarette would be the outfit I would wear to the bar, but it’s not the outfit I would wear to school.” (Johnson et al., 2003, p. 393)	Purposefully shifting their smoker identities context to context (81, 83, 127, 143)	12. There is a possibility for a shift between smoker identities	
“When I see my future, I don’t see myself as a smoker, I don’t see myself even as a social smoker.” (Hoek, Maubach et al., 2013, p. 263)	Identification with future smoker or nonsmoker identities (11, 12, 15, 33, 93, 114)	13. Future smoker identities exist	
“A big chunk of my life away and I might not want to go about with the same pals and not being able to do that would be horrible.” (Amos et al., 2005, p. 185)	Concern about losing the social image and social benefits of being a smoker in case of quitting smoking (4, 14, 17, 76, 77, 87, 94)	14. Smoker identities influence making a quit attempt	IV. Behavior in relation to smoking
“Like, I knew consciously that I didn’t want to be a smoker.” (Johnson et al., 2003, p. 391)	Having clear intention to distance oneself from being a smoker can help a person abstain from smoking (69, 73, 79, 124, 125, 126, 127, 128)	15. Smoker identities influence long-term abstinence
