# Supplementary material for: Smoker Identity and Its Potential Role in Young Adults’ Smoking Behavior: A Meta-Ethnography
Source: Health Psychol. 2015 Jan 26;34(10):992–1003. doi: 10.1037/hea0000191 (PMC4577249; doi:10.1037/hea0000191)
Supplement: Supplementary file 1 [file hea-HEA-2014-2148-Metaethnography_acceptedHealthPsychology_onlinesuppl.doc]

Table S1: Study characteristics of the included papers

| **Authors** | **Aims** | **Design** | **Setting** | **Target population** | **Sample size** | **Participants’ age** | **Gender**  **(Female)** | **Ethnicity** | **Smoking status** |
| --- | --- | --- | --- | --- | --- | --- | --- | --- | --- |
| Amos et al., 2006 | To explore participants' understanding of their smoking, their attitudes towards quitting and cessation support and how issues around quitting are linked with their social context. | Qualitative interview study | UK | Adolescents | N=99 | 16-19 | 52.5% | White Scottish  (100%) | Daily smokers (n=75);  Occasional smokers (n=24) |
| Berg et al., 2010 | To examine how college students define the term ‘smoker’, what their experiences are with quitting and their motives for and barriers to cessation. | Qualitative focus group study | USA | College and university students | N=73 | 18-25 | 56.2% | White (89.0%) | Smoked 25+ days of past 30 days (n=24) |
| Brown et al., 2011 | To understand the personal motivations and forces that influence college-level occasional smoking. | Qualitative focus groups | USA | University students | N=53 | 18-25 | 56.6% | White  (50.9%) | Occasional smokers (n=53) |
| Gilbert, 2007 | To explore what cigarette smoking means for young women. | Qualitative interview study | Australia | Young women | N=20 | 18-24 | 100% | Australian (100%) | Daily smokers (n=20) |
| Hoek et al., 2013 | To explore how young adult social smokers view and define their smoking and reconcile conflicting smoker and non-smoker identities; what factors facilitate or catalyse social smoking; and what interventions could ameliorate these. | Qualitative interview study | New Zealand | Young adult social smokers | N=13 | 19-25 | 30.8% | New Zealander (76.9%) | Social smokers (n=13) |
| Hoek et al., 2013 | To explore how young adult smokers interpret current tobacco warning messages. | Qualitative interview study | New Zealand | Young adult smokers | N=17 | 18-30 | 47.1% | New Zealander (100%) | Daily smokers (n=9);  Social smokers (n=8) |
| Johnson et al., 2003 | To explore how youth describe their tobacco use and identify the central narratives. | Qualitative interview study | N.R. | Young people | N=35 | 14-18 | 51.4% | N.R. | Ever smokers, occasional and daily smokers |
| Kishchuk et al., 2004 | To explore students' views on smoking cessation interventions. | Qualitative focus groups | Canada | College students | N=69 | 18-34 | 43.5% | N.R. | Smokers (69.6%);  Ex-smokers (26.1%) |
| Lawson, 1994 | To explore why low-income pregnant smokers continue to smoke and what are the perceived benefits of tobacco use as well as what believes influence their cessation of smoking. | Qualitative interview study | N.R. | Low-income pregnant adolescents | N=20 | 16-18 | 100% | White (70.0%) | Daily smokers (100%) |
| Lennon et al., 2005 | To explore personal and social factors influencing the likelihood of smoking among young women. | Qualitative focus group and  interview study | Australia | Young women | N.R. | 16-28 | 100% | N.R. | Smokers (5 focus groups, 3 interviews); Non-smokers (6 focus groups, 1 interview); Smokers and non-smokers (3 focus groups, 2 interviews) |
| MacFadyen, 2003 | To examine the impact of smoking related imaginary in youth magazines on young smokers. | Qualitative focus group study | UK | College and university students | N.R. | 17-18 | Females (6 focus groups),  Males (6 focus groups) | N.R. | Regular smokers (6 focus groups); Occasional smokers (6 focus groups) |
| Moffat et al., 2001 | To explore the meaning of nicotine addiction for teenage girls in the context of their lives and smoking patterns. | Qualitative interview study | N.R. | Adolescents | N=12 | 14-17 | 100% | N.R. | Ever smokers |
| Rooke et al., 2013 | To explore the role of smoking in the spaces of the night-time economy, how the smokefree legislation impacted on young adults, how they managed their smoker identity in different spaces and situations in relation to the negative social climate around smoking and how it affected their social identity. | Qualitative interview study | England | Young adult smokers and ex-smokers | N=27 | 18-29 | 44.4% | White British (51.9%) | Daily and occasional smokers |
| Scheffels et al., 2007 | To explore how young smokers talk about continuing to smoke in the context of increasing structural and symbolic pressure to quit, issues of legitimacy, meaning and identity. | Qualitative interview study | Norway | Young adult smokers | N=21 | 18-23 | 47.6% | N.R. | Daily smokers (n=19); Occasional smoker (n=2) |
| Scheffels, 2008 | To explore young adult smokers' construction of meaning and identity in accounts of cigarette brands and package design. | Qualitative interview study | Norway | Young adult smokers | N=21 | 18-23 | 47.6% | N.R. | Daily smokers (n=19); Occasional smoker (n=2) |
| Scheffels, 2009 | To explore young adult smokers' experiences with smoking in relation to the construction of identity. | Qualitative interview study | Norway | Young adult smokers | N=21 | 18-23 | 47.6% | N.R. | Daily smokers (n=19); Occasional smoker (n=2) |
| Wiltshire, 2005 | To explore young smokers' experiences and attitudes towards smoking, their understandings of being a smoker and the role smoking plays in their lives. | Qualitative interview study | Ireland | Young people | N=99 | 16-19 | 52.5% | White Scottish  (100%) | Daily smokers (n=75);  Occasional smokers (n=24) |

**References**

1. Amos A, Wiltshire S, Haw S, McNeill A. Ambivalence and uncertainty: experiences of and attitudes towards addiction and smoking cessation in the mid-to-late teens. Health Education Research. 2006;21(2):181-91.

2. Berg CJ, Parelkar PP, Lessard L, Escoffery C, Kegler MC, Sterling KL, et al. Defining "smoker": College student attitudes and related smoking characteristics. Nicotine & Tobacco Research. 2010;12(9):963-9.

3. Brown AE, Carpenter MJ, Sutfin EL. Occasional smoking in college: who, what, when and why? Addictive Behaviors. 2011;36(12):1199-204.

4. Gilber E. Constructing 'Fashionable' Youth Identities: Australian Young Women Cigarette Smokers. Journal of Youth Science. 2007;10(1):1-15.

5. Hoek J, Maubach N, Stevenson R, Gendall P, Edwards P. Social smokers' management of conflicting identities. Tobacco Control 2013;22(4):261-5.

6. Hoek J, Hoek-Sims A, Gendall P. A qualitative exploration of young adult smokers' responses to novel tobacco warnings. BMC Public Health. 2013;13(609).

7. Johnson JL, Lovato CY, Maggi S, Ratner PA, Shoveller J, Baillie L, et al. Smoking and Adolescence: Narratives of Identity. Research in Nursing & Health. 2003;26:387-97.

8. Kishchuk N, Tremblay M, Lapierre J, Heneman B, O'Loughlin J. Qualitative investigation of young smokers' and ex-smokers' views on smoking cessation methods. Nicotine & Tobacco Research. 2004;6(3):491-500.

9. Lawson EJ. The role of smoking in the lives of low-income pregnant adolescents: a field study. Adolescence. 1994;29(113):61-79.

10. Lennon A, Gallois C, Owen N, McDermott L. Young Women as Smokers and Nonsmokers: A Qualitative Social Identity Approach. Qualitative Health Research. 2005;15(10):1345-59.

11. MacFadyen L, Amos A, Hastings G, Parkes E. 'They look like my kind of people' - perceptions of smoking images in youth magazines. Social Science & Medicine. 2003;56:491-9.

12. Moffat BM, Johnson JL. Through the Haze of Cigarettes: Teenage Girls' Stories About Cigarette Addiction. Qualitative Health Research. 2001;11(5):668-81.

13. Rooke C, Amos A, Highet G, Hargreaves K. Smoking spaces and practices in pubs, bars and clubs: Young adults and the English smokefree legislation. Health & Place. 2013;19:108-15.

14. Scheffels J, Schou KC. To be one who continues to smoke: Construction of legitimacy and meaning in young adults' accounts of smoking. Addiction Research and Theory. 2007;15(2):161-76.

15. Scheffels J. A difference that makes a difference: young adult smokers' accounts of cigarette brands and package design. Tobacco Control. 2008;17:118-22.

16. Scheffels J. Stigma, or sort of cool. European Journal of Cultural Studies. 2009;12(4):469-86.

17. Wiltshire S, Amos A, Haw S, McNeill A. Image, context and transition: smoking in mid-to-late adolescence. Journal of Adolescence. 2005;28:603-17.

Table S2: List of excluded papers with reason for exclusion after full-text screening

| **Author, year** | **Reason for exclusion** |
| --- | --- |
| Afifi Soweid et al., 2004 | Religious identity: This quantitative study assessed the extent to which people’s religious identity is associated with their smoking behaviour. |
| Alexander et al., 2010 | Gender identity: This study explored young people’s views on gender identities and smoking behaviour. |
| Aloise-Young & Hennigan, 1996 | The paper reports quantitative evidence on smoker identity; participants’ age: 5^th^-8^th^ graders |
| Aloise-Young & Graham, 1996 | The paper reports quantitative evidence on smoker identity; participants’ age: 5^th^-8^th^ graders |
| Amos et al., 2007 | Gender identity: The study explored young people’s (age 15-16) views on their gender identity and the meanings they attach to their smoking. |
| Amos et al., 1998 | The paper reports quantitative evidence on smoker identity; participants’ age: age 12-19 |
| Amos et al., 1997 | The paper reports quantitative evidence on smoker identity; participants’ age: age 12-19 |
| Angstman et al., 2009 | Ethnic identity: The study assessed the relationship between identification as an American Indian and smoking behaviour. |
| Asbridge et al., 2005 | Ethnic identity: The study assessed the relationship between ethnic identity and smoking behaviour in young people (age 13-19). |
| Aycan et al., 1998 | Ethnic identity: The study assessed the relationship between acculturation and smoking behaviour. |
| Barger, 2008 | Ethnic identity: This quantitative study assessed the relationship between ethnic identity and smoking behaviour. |
| Berg et al., 2009 | The paper reports quantitative evidence on smoker identity. |
| Biddle et al., 1985 | The paper reports quantitative evidence on smoker identity; participants’ age: age 12-18 |
| Bland et al., 1975 | The paper reports quantitative evidence on smoker identity; participants’ age: age 10-11 |
| Bottorff et al., 2000 | Participants’ age: age 18-39 |
| Bottorff et al., 2006 | Participants’ age: age 20-49 |
| Brook et al., 2010 | Ethnic identity: This quantitative study assessed the relationship between young African-Americans’ and Puerto Ricans’ views on their ethnic identity and smoking behaviour over time. |
| Burton et al., 1989 | Participants’ age: 7^th^ graders |
| Cassidy, 2006 | Smoker identity was not investigated beyond the assessment of smoking status: This qualitative study assessed the relationship between perceptions of mobile phone use in the context of social identity and adolescent smoking. |
| Castro et al., 2009 | Ethnic identity: This quantitative study assessed the influence of aspects of cultural identity on self-efficacy and the perceived benefits of smoking. |
| Chae et al., 2008 | Ethnic identity: This quantitative study assessed the relationship between self-report of ethnic specific discrimination with current smoking. |
| Chang, 2007 | Smoker identity was not investigated beyond the assessment of smoking status: The study assessed the congruency between ideal self-images of smokers and non-smokers and the perceived images of smokers portrayed in cigarette advertisements. |
| Chassin, 1981 | Smoker identity was not investigated beyond the assessment of smoking status: The study assessed the relationship between self-perceptions, perceptions of stereotypic male/female smokers/non-smokers and the association with intention to smoke. |
| Choi et al., 2010 | The paper reports quantitative evidence on smoker identity. |
| Cooper, 1989 | Smoker identity was not investigated beyond the assessment of smoking status: The study assessed the social image of the young female smokers in female smoker and non-smoker college students. |
| Dal Cin et al., 2007 | Smoker identity was not investigated beyond the assessment of smoking status: The study examined whether identification with a smoker character increases the implicit associations between self and smoking. Half of the participants were never smoker. |
| Davey et al., 2012 | Participants’ age: age 12-13. |
| Denscombe, 2001 | Participants’ age: age 15-16 |
| Eiser et al., 1978 | Smoker identity was not investigated beyond the assessment of smoking status. The paper reports quantitative evidence in participants aged 18-54. |
| Eiser et al., 1977 | Smoker identity was not investigated beyond the assessment of smoking status: The study assessed smokers’ and non-smokers’ attitudes towards cigarette smoking and their perceptions of smokers. |
| Elkind, 1985 | Gender identity: The study explored the social interpretations of female smoking behaviour. |
| Epstein et al., 1998 | Ethnic identity: The study assessed the relationship between Hispanic acculturation and smoking behaviour. |
| Evans et al., 1990 | Gender identity: The study assessed the relationship between being characterized as androgynous in sex-role terms and smoking behaviour. |
| Evans et al., 2006 | Smoker identity was not investigated beyond the assessment of smoking status: The study assessed the relationship between social images of smokers, social environment and adolescent smoking. |
| Falomir et al., 1999 | The paper reports quantitative evidence on smoker identity. |
| Falomir-Pichastor et al., 2007 | The paper reports quantitative evidence on smoker identity; participants’ age: age 11-15 |
| Farrimond et al., 2010 | Participants’ age: age 20-59 |
| Fidler & West, 2009 | The paper reports quantitative evidence on smoker identity; participants’ age: age 16-65+ |
| Freeman et al., 2001 | The paper reports quantitative evidence on smoker identity. |
| Friebely et al., 2013 | The paper reports quantitative evidence on smoker identity. |
| Fuqua et al., 2012 | Smoker identity was not investigated beyond the assessment of smoking status: The study assessed the relationship between multiple peer group self-identification and smoking behaviour. |
| Gerrard et al., 2005 | Smoker identity was not investigated beyond the assessment of smoking status: The study assessed the relationship between children's images of smokers and the onset of smoking. |
| Gilbert, 2007 | Gender identity: The study explored whether smoking constitutes part of women’s gender identity. |
| Grube et al., 1984 | Smoker identity was not investigated beyond the assessment of smoking status: The study assessed the similarity between value images of smokers and non-smokers. |
| Harris et al., 2008 | The paper reports quantitative evidence on smoker identity. |
| Hassandra et al., 2011 | Participants’ age: age 10-18; mostly non-smokers |
| Hertel et al., 2012 | The paper reports quantitative evidence. |
| Hoek et al., 2012 | The study explored young adults’ perceptions about images associated with tobacco brands and possible effects of plain packaging on the social meaning of cigarette brands. More than half of the participants were non-smokers and it is not evident whether they were ever smokers. |
| Hoek et al., 2011 | The paper reports quantitative evidence on smoker identity in terms of adult smokers’ and non-smokers’ (age 12-24) identification with role models in anti-tobacco campaigns. |
| Hoie et al., 2010 | The paper reports quantitative evidence on smoker identity. |
| Horneffer-Ginter, 2008 | Smoker identity was not investigated beyond the assessment of smoking status: The paper addressed the topic of possible selves in relation to smoking. |
| Ioannou, 2010 | Participants’ age: age 15-17. |
| Koblitz et al., 2009 | The paper reports quantitative evidence on smoker identity. |
| Kong et al., 2012 | Ethnic identity: The study assessed the relationship between aspects of ethnical identity and smoking behaviour. |
| Lee, 1989 | Smoker identity was not investigated beyond the assessment of smoking status: The study assessed the images of male and female smokers held by students as a function of their smoking status. |
| Lee et al., 2011 | Participants’ age: age 12-16. |
| Lee et al., 2013 | The paper reports quantitative evidence on smoker identity. |
| Legge Muilenburg et al., 2006 | Participants’ age: children at middle school age |
| Levinson et al., 2007 | The paper reports quantitative evidence on smoker identity. |
| Lloyd et al., 1997 | Participants’ age: age 11-16. |
| Lucas & Lloyd, 1999 | Participants’ age: age 11-16. |
| Mao et al., 2013 | The study explored female non-smokers’ views on male smoking in China. |
| McCool et al., 2003 | Smoking identity was not investigated: The study explored adolescents’ interpretation of smokers' images in popular films; participants’ smoking status was not assessed. |
| McCool et al., 2013 | More than half of the sample consisted of non-smokers and it was not reported whether they were ever smokers at the time of enrolment. |
| McInman, 1991 | Smoker identity was not investigated beyond the assessment of smoking status: The study assessed multiple dimensions of self-concept in relation to smoking. |
| McKennell, 1969 | Smoker identity was not investigated beyond the assessment of smoking status: The study assessed adolescents' ratings regarding their views on 'the boy-smoker', 'the boy non-smoker', 'the self' and 'the ideal self' on a 19-item bipolar scale to conduct factor analysis. |
| Mermelstein, 1999 | Participants’ age: age 11-19. |
| Michell, 1997 | Participants’ age: age 11-13. |
| Moan & Rise, 2005 | The paper reports quantitative evidence on smoker identity. |
| Moan & Rise, 2006 | The paper reports quantitative evidence on smoker identity; participants’ age: age 13-14 |
| Moran et al., 2012 | Smoker identity was not investigated beyond the assessment of smoking status: The study assessed the relationship between young people’s identification with different social groups and the effects of antitobacco campaign. |
| Mosbach, 1988 | Smoker identity was not investigated beyond the assessment of smoking status: The study assessed the relationship between peer group identification and smoking. |
| Nguyen et al., 2012 | Ethnic identity: The study assessed the influence of experiences of racial discrimination and ethnic identity on prenatal smoking. |
| Nichter et al., 2008 | Participants’ age: age 18-43. |
| Nichter et al., 2007 | Participants’ age: age 18-43. |
| Odgen et al., 1997 | The paper reports quantitative evidence on smoker identity. |
| Okoli et al., 2011 | The paper reports quantitative evidence on smoker identity; participants’ age: age 12-19. |
| Okoli et al., 2008 | The paper reports quantitative evidence on smoker identity; participants’ age: age 12-19. |
| Oliffe et al., 2012 | Smoker identity was not assessed: The study explored men’s perspectives on gender-sensitive health promotion programmes. |
| Parker et al., 1998 | Ethnic identity: The study assessed the relationship between ethnic identity as an African American or a Latino and smoking behaviour. |
| Phua, 2013 | The study reports quantitative evidence: The study assessed the whether identification with reference social groups moderates the relationship between group norms and smoking cessation self-efficacy. |
| Piko & Gibbons, 2007 | Smoker identity was not investigated beyond the assessment of smoking status: The study assessed adolescents' views on smoker prototypes as a function of smoking status. |
| Plumridge et al., 2002 | Participants’ age: age 13-14. |
| Remafedi et al., 2008 | Sexual identity: The study assessed the relationship between different sexual identities and smoking behaviour. |
| Ridner et al., 2010 | The paper reports quantitative evidence on smoker identity. |
| Ritchie et al., 2010 | Participants’ age: age 18-60+. |
| Rugkasa et al., 2003 | Gender identity: The study explored young people’s perceptions of gender identity and smoking. |
| Scheffels & Lund, 2005 | Smoker identity was not investigated beyond the assessment of smoking status: The paper addressed the topic of smoking motivations and confidence in ability to stop smoking as a function of smoking status. |
| Schofield et al., 2001 | Smoker identity was not investigated beyond the assessment of smoking status: The study assessed the relationship between favourable smoking norms in the peer group and smoking behaviour. |
| Shadel & Tharp-Taylor, 2009 | Smoker identity was not investigated beyond the assessment of smoking status: The study assessed adolescents’ (age 11-17) identification with role models in anti-tobacco messages. |
| Shadel & Abrams, 2004 | The study assessed never smokers’ identification with smokers in cigarette advertisements. |
| Shadel & Abrams, 2004 | The study assessed the relationship between self-conflicts and the identification with smokers from cigarette advertisements in never smokers. |
| Shadel et al., 2008 | The study assessment the relationship between identification with smokers from cigarette advertisements, aspects of self- concept and intention to smoke in never smokers. |
| Shadel et al., 2009 | The study assessed the relationship between exposure to cigarette advertisements and intention to smoke in never smokers. |
| Song et al., 2013 | Smoker identity was not investigated beyond the assessment of smoking status: The study assessed the association between exposure to possible future selves by using avatars in computer games and attitudes toward smoking. |
| Soweid & Salem, 2004 | Religious identity: The study assessed the relationship between religious identity and smoking behaviour. |
| Spijkerman et al., 2005 | Smoker identity was not investigated beyond the assessment of smoking status: The study assessed the relationship between adolescents' perceptions of smoker prototypes and smoking onset. |
| Sussman et al., 1994 | Smoker identity was not investigated beyond the assessment of smoking status: The study assessed the relationship between identification with social groups and smoking behaviour, but the analysis did not go beyond assessing smoking status (i.e. did not assess smoker identity). |
| Thompson et al., 2009 | Participants’ age: age 20-73. |
| Tracy et al., 2012 | The paper reports quantitative evidence on smoker identity. |
| Treacy et al., 2007 | Participants’ age: age 11-16. |
| Vahey et al., 2010 | Smoker identity was not investigated beyond the assessment of smoking status: An implicit association test was conducted with social identity and smoking. |
| van den Putte et al., 2009 | Participants’ age: age 16-70. |
| Van der Heiden et al., 2013 | Participants’ age: age 18-48. |
| Wolsko et al., 2009 | Ethnic identity: The study assessed the relationship between cultural identity as a Yup’ik and smoking behaviour. |
| Young, 1993 | Participants’ age: age 13-18. |

Table S3: List of first order interpretations

| **ID of key constructs** | **First order interpretations** | **Number of occurrences** | **Number of studies reporting** |
| --- | --- | --- | --- |
| 89 | Identification with a social/casual smoker identity, rather than with a smoker identity. | 7 | 7 |
| 67 | Being a smoker to gain social benefits (e.g. to help socializing, provide feelings of being included). | 7 | 6 |
| 3 | Being a smoker means you purchase cigarette on your own rather than borrowing it from others; if not, then not being a smoker. | 6 | 5 |
| 18 | Being a smoker means you smoke more often; if not, then not being a smoker. | 6 | 5 |
| 35 | To avoid stigma consciously choose where, when and with whom they smoke or hide being a smoker. | 5 | 5 |
| 110 | Not being a smoker due to perceived control over smoking. | 5 | 5 |
| 46 | Identity conflicts due to smoking, because it diminishes current or future identity aspirations. | 5 | 4 |
| 139 | Being a smoker is an unattractive identity due to stigma and negative social discourse associated with it. | 5 | 4 |
| 20 | Not having a smoker identity, but smoking when drinking. | 4 | 4 |
| 44 | Smoking to express an identity, which is in accordance with what is valued by the social group to maintain social status. | 4 | 4 |
| 116 | Having a non-smoker identity despite smoking cigarettes. | 4 | 4 |
| 14 | Concern about loosing the social image and social benefits of smoking in case of quitting. | 4 | 3 |
| 22 | Denial of being a smoker is context dependent; thus, maintaining non-smoker image in front of significant others (e.g. family, employers, strangers), but engaging in smoking with friends. | 4 | 3 |
| 82 | Smoker identity is not a binary construct, rather multiple smoker identities coexist. | 4 | 3 |
| 26 | Being a smoker means that smoking involves a large part of life (e.g. need to smoke at certain times), part of daily routine; if not, then not being a smoker. | 4 | 2 |
| 4 | Identification as a non-smoker undermines desire to quit smoking. | 3 | 3 |
| 6 | Social smokers want to dissociate negative images of an addicted smoker from themselves | 3 | 3 |
| 21 | Identity self-label as 'only social smoker' to justify smoking. | 3 | 3 |
| 93 | Identification with future smoker or non-smoker identities. | 3 | 3 |
| 107 | Addicted (daily) smokers have negative social image and are looked down by non-daily smokers. | 3 | 3 |
| 111 | Not being a smoker due to smoking less than others (even though a person smokes 5-15 CPD). | 3 | 3 |
| 113 | Not being a smoker due to perceived ease of quitting smoking. | 3 | 3 |
| 120 | Being a smoker means that one is addicted to cigarette and experiences cravings; if not, then not being a smoker. | 3 | 3 |
| 88 | Smoker identity is not part of identity. | 3 | 2 |
| 103 | Choosing a specific cigarette brand to make a statement of one's identity. | 3 | 2 |
| 135 | Don’t want to be seen as a smoker and feelings of guilt and regret are associated with it. | 3 | 2 |
| 75 | Negative feelings and self-portrayal due to being a smoker. | 3 | 1 |
| 9 | Rejection of being addicted serves as a tool to maintain identity as a person in control and smoking at the same time. | 2 | 2 |
| 23 | Smoking to appear someone who is cool and to rebel against good girl identity. | 2 | 2 |
| 27 | Negative physical (e.g. having yellow teeth, smelling) and psychological characteristics (e.g. anxious) are associated with being a smoker. | 2 | 2 |
| 28 | Not a smoker because would not smoke alone. | 2 | 2 |
| 31 | Being an addicted smoker is a feared identity. | 2 | 2 |
| 40 | Smoking allows being part of the cool group. | 2 | 2 |
| 63 | Identification with a non-daily/occasional smoker identity as opposed to a smoker identity. | 2 | 2 |
| 72 | Considerate and inconsiderate smoker identity: Being a considerate smoker is better/superior than being inconsiderate (exposing others to smoking e.g. in pregnancy). | 2 | 2 |
| 83 | Smoker identity is not a static construct, because there can be a shift between identities. | 2 | 2 |
| 84 | Central smoker identity can be identified. | 2 | 2 |
| 90 | Being a smoker is seen as something that could be worse by being a pack-a-day smoker, a drug user or an alcoholic. | 2 | 2 |
| 94 | Being a smoker does not match with other valued identity aspects (e.g. be a good mother, successful), and this motivates making a quit attempt. | 2 | 2 |
| 108 | Cool smoker identity: Perceived controlled over smoking is part of the identity and image as a cool smoker as opposed to addicted smokers. | 2 | 2 |
| 112 | Not being a smoker because cravings are related to the social aspects of smoking. | 2 | 2 |
| 119 | The realization that someone is a smoker is associated with an unsuccessful quit attempt. | 2 | 2 |
| 73 | Being vulnerable to be a smoker again due to perceived benefits of smoking. | 2 | 1 |
| 101 | Choosing a specific cigarette brand to express group identity. | 2 | 1 |
| 105 | Choosing a specific cigarette brand to express and upgrade one's social class. | 2 | 1 |
| 117 | Rules regarding smoking are different when alcohol is involved. | 2 | 1 |
| 126 | Being vulnerable to smoke again because reasons for not smoking were situational, contextual and influenced by significant others. | 2 | 1 |
| 127 | Having clear intention to distance themselves from smoker self and smoking, and consciously forming a plan and belief how not to be a smoker again can help individuals to abstain from smoking. | 2 | 1 |
| 135 | Do not want to be seen as a smoker. | 2 | 1 |
| 142 | Being a smoker means that you can be looked down on and get labelled. | 2 | 1 |
| 1 | Regarding oneself as a smoker depends on whether one thinks one is addicted or smokes due to habit. | 1 | 1 |
| 2 | Being addicted means that someone smokes every day; if not, then not being a smoker | 1 | 1 |
| 5 | Being addicted is associated with negative images and feelings (e.g. being desperate, having cravings). | 1 | 1 |
| 7 | Ambivalence whether they see themselves as addicted. | 1 | 1 |
| 8 | Thoughts about whether someone is addicted are hard to articulate. | 1 | 1 |
| 10 | Perceived control over smoking; thus, not being addicted to smoking. | 1 | 1 |
| 11 | Those accepting being addicted do not hold future non-smoker identity. | 1 | 1 |
| 12 | Not seeing oneself as a non-smoker in the future due to unsuccessful previous quit attempts. | 1 | 1 |
| 13 | Realisation of being addicted is associated with a quit attempt and how hard it was. | 1 | 1 |
| 15 | Being a smoker is part of adult identity, so the person holds future smoker identity and does not want to quit. | 1 | 1 |
| 16 | Not being a smoker because 'never gasping'. | 1 | 1 |
| 17 | Constructing a smoker identity that is based on being informed about the health risks undermines any desire to quit smoking. | 1 | 1 |
| 19 | Smoking communicates that a person is fun loving and takes pleasure over health awareness. | 1 | 1 |
| 24 | Being a smoker means you started smoking long ago; if not, then not being a smoker. | 1 | 1 |
| 25 | Being a smoker to be seen as a more relaxed person in the future. | 1 | 1 |
| 29 | Being a smoker is an unattractive identity; therefore, want to stop. | 1 | 1 |
| 30 | Do not smoke every day due to the importance of self-appearance. | 1 | 1 |
| 32 | Dependence is a negative trait that distinguishes smokers from occasional smokers. | 1 | 1 |
| 33 | Formulating personal non-smoking rules around life events: 'Would not smoke if had a child'. | 1 | 1 |
| 34 | Being a smoker does not cause dissonance with other identities and feeling of stigma when drinking with others. | 1 | 1 |
| 36 | Being a smoker is cool with other smokers but something that needs to be hidden in front of non-smokers (dual image of smoking). | 1 | 1 |
| 37 | Smoking to increase self-confidence in public situations. | 1 | 1 |
| 38 | Being a smoker is a statement of glamour. | 1 | 1 |
| 39 | Smoking to construct self-image of a sophisticated person. | 1 | 1 |
| 41 | Smoking to look sexy. | 1 | 1 |
| 42 | Smoking to achieve an ideal self. | 1 | 1 |
| 43 | Smoking to form a mature identity. | 1 | 1 |
| 45 | Demarcation strategies to avoid smoker identity. | 1 | 1 |
| 47 | Smoking socially is a short-term indulgence, while addicted smokers smoke permanently. | 1 | 1 |
| 48 | Smoking to look more elegant. | 1 | 1 |
| 49 | Being successful is an aspired future identity, which is conflicting with being a smoker in the future. | 1 | 1 |
| 50 | Immediate social acceptance from the group and disapproval from the wider context creates tension. | 1 | 1 |
| 51 | Do not enjoy smoking or anticipate smoking situations, but this is a price to be paid for social acceptance. | 1 | 1 |
| 52 | Consuming alcohol liberates smokers from their non-smoker identity and reconcile dissonance what normally would keep them away from smoking. | 1 | 1 |
| 53 | Internal conflicts due to dual smoker identities were managed by reducing the salience of these conflicts. | 1 | 1 |
| 54 | Performative smoker identity: mainly related to smoking initiation. | 1 | 1 |
| 55 | Internal conflicts due to perceived superior status as a non-smoker but engaging with a stigmatized behaviour. | 1 | 1 |
| 56 | Construct a smoker identity as a defensive community against stigmatization. | 1 | 1 |
| 57 | Rationalisation (i.e. smoking is not a rational choice when consuming alcohol) to maintain dual identities as a non-smoker who smokes. | 1 | 1 |
| 58 | Formulating personal smoking rules around alcohol consumption, such as 'I don't smoke until I get drunk", to manage internal conflicts. | 1 | 1 |
| 59 | Perceived benefits of being in a group outreached the risk they are aware of taking with continuing smoking. | 1 | 1 |
| 60 | Defensive mechanism: being a smoker is a rational decision and the risks are within the acceptable range. | 1 | 1 |
| 61 | Smoking is part of identity as a young person. | 1 | 1 |
| 62 | Smoking is perceived as something that comes with certain benefits. | 1 | 1 |
| 64 | Identification with a defensive or negotiating smoker identity. | 1 | 1 |
| 65 | Having a vulnerable non-smoker identity associated with feelings of desire to smoke due to social pressure. | 1 | 1 |
| 66 | Having a confident non-smoker identity means you tried smoking, smoked for a while, but then stopped for good. | 1 | 1 |
| 68 | Having an ardent non-smoker identity means the person completely rejects tobacco and smokers. | 1 | 1 |
| 69 | Being someone who could quit and does not want to smoke any more gives strengths and feeling of coolness. | 1 | 1 |
| 70 | Having an accepting non-smoker identity means you do not see yourself as being vulnerable to smoking and therefore do not mind to be around smokers. | 1 | 1 |
| 71 | Those with an in-control smoker identity see themselves as smokers but only in certain situations. | 1 | 1 |
| 74 | For confirmed smokers, smoker identity is something that is adopted by time ('becoming a smoker') and acceptance of smoker identity seems to be unquestioned. | 1 | 1 |
| 76 | Those with a strong smoker identity have vague plans about how to stop in the future. | 1 | 1 |
| 77 | Those with a strong smoker identity claim they cannot be pressured to quit unless they want to. | 1 | 1 |
| 78 | Contrite smokers: societal messages prompted smokers to feel regret for their habit. They do not want to be smokers and do not want to be seen as smokers. | 1 | 1 |
| 79 | Need to strongly believe to be able to be a non-smoker to quit smoking for good. | 1 | 1 |
| 80 | External regulations in social context would be needed to reassert non-smoker identity and diminish identity conflict (i.e. being a smoker vs. non-smoker). | 1 | 1 |
| 81 | Having multiple smoker identities does not necessarily cause tension, rather let people act context dependent. | 1 | 1 |
| 85 | Different agents underlie different smoker identities. | 1 | 1 |
| 86 | Confident non-smokers have active agent in the creation of their smoker identity, whereas others portrayed themselves as passive without choice or control. | 1 | 1 |
| 87 | Intervention that increases incoherence between self-image and smoking is seen to motivate smoking cessation. | 1 | 1 |
| 91 | Being a smoker gives feeling of superiority above other substance users. | 1 | 1 |
| 92 | Being a smoker is a relief because it helps not being involved in criminal acts. | 1 | 1 |
| 95 | Smoker identity is something that is performed and also that is experienced (becoming). | 1 | 1 |
| 96 | Rationalization why not being a smoker evolves over time and the definition of smokers always serves one's interest as not being seen oneself as a smoker. | 1 | 1 |
| 97 | Smoking means being and becoming someone as well as being with someone. | 1 | 1 |
| 98 | The realization of being addicted means the construction of smoker identity. | 1 | 1 |
| 99 | Smoking as an identity trademark. | 1 | 1 |
| 100 | Unanticipated addiction among social smokers creates identity conflict between being a smoker and other lifestyle choices (e.g. being an athlete). | 1 | 1 |
| 102 | Choosing a specific cigarette brand to negotiate a more individual identity. | 1 | 1 |
| 104 | Choosing a specific cigarette brand to express an identity that refers to geographic locality. | 1 | 1 |
| 106 | Being addicted means that one experiences the need to have a cigarette; if not, then not being a smoker. | 1 | 1 |
| 109 | In an attempt to avoid becoming addicted, one is controlling oneself in terms of when and how many cigarettes he/she smokes. | 1 | 1 |
| 114 | Not seeing oneself as a non-smoker in the future due to strong need to smoke. | 1 | 1 |
| 115 | The realisation that someone is addicted is associated with smoking not being social any more and not confining to particular places and context. | 1 | 1 |
| 118 | The realization that someone is a 'proper smoker' is associated with buying one's own cigarette. | 1 | 1 |
| 121 | Choosing light cigarettes and feminine brand to express gender identity as a women. | 1 | 1 |
| 122 | Negative health effects are not seen as personally relevant due to being an occasional smoker. | 1 | 1 |
| 123 | Multiple dimensions of smoker identities exist. | 1 | 1 |
| 124 | Being vulnerable to smoke again due to stopping was not grounded in strong personal conviction about the need to avoid or abstain from smoking. | 1 | 1 |
| 125 | Having ‘not a complete non-smoker identity’ opens the possibility for smoking in the future. | 1 | 1 |
| 128 | Never wanted to take on the smoker identity and knew consciously that did not want to be a smoker helped abstain from smoking. | 1 | 1 |
| 129 | Accepting smoker identity and the realization of all the negative health effects, costs and the effects smoking has on appearance. | 1 | 1 |
| 130 | Smokers tend to be looked as ‘stupid’ by others; therefore, smoking is not something participants were proud of despite enjoy it. | 1 | 1 |
| 131 | Concerns about moving between smoking and non-smoking spaces (e.g. going out to have a cigarette; then coming back into the bar) related to bodily signs of being a smoker (e.g. the smells associated with it). | 1 | 1 |
| 132 | Not being able to smoke inside requires a greater need to manage smoking in relation to presentation of self. | 1 | 1 |
| 133 | The rearrangement of smoking space (e.g. introducing outdoor spaces, beer gardens) allows young smokers to produce a positive, fun and sociable smoker identity. | 1 | 1 |
| 134 | Smoking is shaping smokers’ night time selves and activities. | 1 | 1 |
| 136 | Smoking is not considered as an acceptable part of one’s professional self; thus, hiding being a smoker in front of customers and colleagues. | 1 | 1 |
| 137 | Smokers belong to social groups with high smoking prevalence; thus, smoking represents a badge of group membership and contributes to social identity. | 1 | 1 |
| 138 | Smokers experienced external disapproval; thus, modified their behaviour to avoid others’ judgement. | 1 | 1 |
| 141 | Being a smoker to look cool and to express a cultural identity of rebellion and notoriety. | 1 | 1 |
| 140 | Identification with a group of smokers as opposed to the conformist non-smokers group. |  |  |
| 143 | Purposefully shifting their identities context to context. | 1 | 1 |

Table S4: List of identified smoker identities

| Social/casual smoker identity |
| --- |
| Non-daily/occasional smoker identity |
| Contrite smoker identity |
| Confirmed smoker identity |
| In-control smoker identity |
| Cool smoker identity |
| Addicted smoker identity |
| Performative smoker identity |
| Defensive/negotiating smoker identity |
| Considerate/inconsiderate smoker identity |
| Non-smoker identity despite smoking |
| Vulnerable non-smoker identity |
| Confident non-smoker identity |
| Ardent non-smoker identity |
| Accepting non-smoker identity |
